# Retrospective analysis of bacterial colonization of necrotic bone and antibiotic resistance in 98 patients with medication-related osteonecrosis of the jaw (MRONJ)

**DOI:** 10.1007/s00784-020-03595-9

**Published:** 2020-10-02

**Authors:** Florian Ewald, Falk Wuesthoff, Robert Koehnke, Reinhard E. Friedrich, Martin Gosau, Ralf Smeets, Holger Rohde, Alexandre T. Assaf

**Affiliations:** 1 Department of General, Visceral and Thoracic Surgery, University Medical Center Hamburg Eppendorf, University of Hamburg, Hamburg, Germany; 2 Department of Oral and Maxillofacial Surgery, University Medical Centre Hamburg Eppendorf, University of Hamburg, 20246 Hamburg, Germany; 3 Department of Oral and Maxillofacial Surgery, Division of Regenerative Orofacial Medicine, University Medical Centre Hamburg Eppendorf, University of Hamburg, Hamburg, Germany; 4 Department of Medical Microbiology, Virology and Hygiene, University Medical Centre Hamburg Eppendorf, University of Hamburg, Hamburg, Germany

**Keywords:** Bacterial colonization, Antibiotic resistance, Medication-related osteonecrosis of the jaw, Antiresorptive drug-induced osteonecrosis of the jaw, ARONJ, MRONJ, Oral microbiota

## Abstract

**Objectives:**

The aim of our study was to describe microbial flora associated with MRONJ and characterize the susceptibility of pathogens to help guide an effective empiric antibiotic treatment in these patients.

**Materials and methods:**

A retrospective, single-center analysis was performed, using 116 bone samples from 98 patients. The bone samples were homogenized and subjected to routine culture methods. Growing bacteria were differentiated to the species level using whole-cell mass spectrometry and subjected to susceptibility testing.

**Results:**

A highly diverse microbial flora was detected in necrotic bone, with a simultaneous presence of two or more bacterial species in 79% of all patients. In at least 65% of samples, gram-negative isolates were detected. Therefore, bacterial species resistant against β-lactamase inhibitors were present in at least 70% of all patients.

**Conclusions:**

The empiric choice of antibiotics in MRONJ patients should consider the high rate of gram-negative bacteria and resistance against β-lactam antibiotics.

**Clinical relevance:**

According to recent guidelines and recommendations, systemic antibiotic treatment is a key component in the treatment of all stage 2 and 3 MRONJ patients. We recommend using fluoroquinolones for empiric treatment and emphasize the use of bacterial cultivation and susceptibility testing to enable an effective antibiotic treatment.

**Electronic supplementary material:**

The online version of this article (10.1007/s00784-020-03595-9) contains supplementary material, which is available to authorized users.

## Introduction

Antiresorptive drug induced- or medication-related osteonecrosis of the jaw (ARONJ, MRONJ) became a serious disease pattern in recent years. The number of patients receiving intravenous (e.g., zoledronate) or oral bisphosphonates (e.g., alendronate) [1] as well as subcutaneous treatment with RANKL inhibitors (e.g., denosumab) or compounds with antiangiogenic effects (i.e., bevacizumab, sorafenib, sunitinib, and others) have been rising over the last decade [2–4]. Indications for antiresorptive drug treatment are non-neoplastic diseases, such as osteoporosis [2], osteitis deformans (Paget’s disease) or arthritis [5], and neoplastic diseases, such as tumor-associated hypercalcemia, multiple myeloma, and skeletal metastases from carcinomas (e.g., breast cancer, renal or prostate cancer) [6–8]. The estimated cumulative incidence of MRONJ in patients receiving bisphosphonates or RANKL inhibitors (e.g., denosumab), human monoclonal antibodies (e.g., bevacizumab), or protein kinase inhibitors (e.g., sorafenib/sunitinib) is thought to be between 0.4 and 21%, depending on the dose and compound used, as well as the route of administration [1, 9, 10].

The diagnostic criteria of MRONJ include an exposure history to bisphosphonates, RANKL inhibitors, or antiangiogenic drugs, exposed bone within the oral cavity, and no history of prior radiation therapy to the jaws [11]. Further affections associated with exposed intraoral necrotic areas are extraoral fistulas, resulting from necrotic bone lesions [1]. Next to a detailed intraoral examination, initial diagnostic procedures routinely include X-ray analysis (e.g., panoramic view, cone beam computed tomography, or computed tomography scans) [12, 13], as well as magnetic resonance imaging (MRI) and scintigraphy [14].

In recent years, it became increasingly evident that bone colonization with bacteria and possibly also fungi plays an important role in the pathogenesis of MRONJ [15]. Healthy bone tissue in the maxilla and mandibula seems to be resistant to microbial colonization, even if exposed to the oral flora. However, in patients treated with antiresorptive or antiangiogenic agents, it is hypothesized that conditions creating an access for bacteria and other pathogens to the vulnerable bone can trigger the development of MRONJ. These conditions include dental procedures, periodontal disease, trauma, or poor-fitting prosthetic devices. In line with this hypothesis, MRONJ predominantly occurs in regions of the body that are exposed to microbial flora like that of the oral cavity [15], whereas MRONJ rarely occurs in other, aseptic regions of the skeletal system.

Although the exact mechanisms still need to be elucidated, there are several hypotheses regarding the mechanisms by which bacterial colonization of infection could induce osteonecrosis. These include the release of acids and proteases, inhibition of bone matrix synthesis, or stimulation of bone degradation [15, 16]. Of note, gram-negative bacteria are hypothesized to play a predominant role in the process by producing toxic products including lipopolysaccharides, directly inducing osteoclast differentiation and activity [15].

Therefore, one of the major aspects in the treatment of MRONJ patients are infections of the adjacent bone and surrounding soft tissues. Especially in MRONJ stages 2 and 3, an effective treatment of bacterial colonization and infection in the affected areas needs to be included into the treatment plan of each patient [16]. The goal of this study was to characterize the composition of colonizing bacteria related to necrotic bone lesions, thereby providing guidance in establishing an effective antibiotic treatment.

## Material and methods

### Patient characteristics

This study is a monocenter, retrospective study. Between June 2016 and September 2018, 98 patients treated at the University Medical Center Eppendorf for clinically and histopathologically confirmed osteonecrosis of the jaw were included in this study (Table 1). The mean age at surgery was 70.9 ± 10.4 years, with a slight predominance of female patients (*n* = 53, 54.1% of all patients). All patients had exposed bone in the oral cavity and were clinically symptomatic (osteonecrosis of the jaw stages 2 or 3, according to the AAOMS position paper on MRONJ from 2014 [17, 18]). MRONJ occurred more often in the mandible than in the maxilla. Of the 98 patients, 55 had received oral or intravenous bisphosphonates, 33 had been treated with denosumab, and 5 patients had received a combination of both. Three patients had no history of antiresorptive drugs but have had intensive chemotherapy for myelodysplastic syndrome (*n* = 2) or metastasized renal cancer (*n* = 1) including antiangiogenic agents [19]. None of the patients had a history of prior radiation therapy of the head or neck region.

**Table 1:** Demographic and clinicopathologic characteristics of study patients

	*n*	%
Male	45	45.9
Female	53	54.1
Age at surgery	70.9	
Underlying diagnosis		
Breast cancer	26	26.5
Prostate cancer	22	22.4
Multiple myeloma	16	16.3
Osteoporosis	15	15.3
Renal cancer	9	9.2
Lung cancer	4	4.1
Aggressive systemic mastocytosis	2	2.0
Other^§^	4	4.1
MRONJ stage at diagnosis		
Stage 2	88	89.8
Stage 3	10	10.2
Localization		
Upper jaw	29	25.0
Lower jaw	75	64.7
Both	12	10.3
Antiresorptive drug regimen		
Bisphosphonate	57	58.2
Denosumab	33	33.7
BP + denosumab	5	5.1
Other^&^	3	3.1
Trigger factor for MRONJ development		
Poor prosthesis fit	26	26,5
Tooth extraction	50	51,0
Unknown	22	22,4

§One patient had metastatic thyroid cancer, chondrosarcoma, and cancer of unknown primary, respectively. One patient was treated for myelodysplastic syndrome

&One patient had received sunitinib, azacitidine, and sorafenib, respectively

Of the 98 patients, 17 patients had either relapsing or recurrent disease. Surgical treatment with resection of necrotic bone with local flap coverage was done twice (*n* = 14) or three times (*n* = 3) in these patients. Therefore, a total of 116 specimens were analyzed.

### Treatment algorithm

After initial evaluations, all patients with clinically and radiologically confirmed diagnosis of stage 2 or 3 MRONJ were treated with oral decontamination using chlorhexidine rinses and oral antibiotics (i.e., amoxicillin and clavulanic acid, 875/125 mg twice daily, in patients with no known drug intolerances against penicillin, clindamycin 300 mg four times daily, or moxifloxacin 400 mg once daily) for 7 days before surgical intervention. Preoperative antibiotic treatment was done using amoxicillin and clavulanic acid in about two-thirds (61%), and moxifloxacin in about one-third of all patients (31%). One day prior to the surgery, all patients turned to inpatient treatment, with intravenous administration of antibiotics, except for moxifloxacin, which was continued orally.

According to the German guideline for the treatment of MRONJ, antibiotic treatment should be continued until signs of local inflammation or bacterial infection of the wound resolve [20, 21]. Therefore, the antibiotic treatment was continued postoperatively. All patients were scheduled for reevaluation and follow up after 1, 2, and 3 weeks. The antibiotic regimen was then adjusted according to the results from cultivation of bone samples and were given until suture removal, usually at days 14 to 21 after surgery.

### Intraoperative sample collection and preparation of microbiological cultures

At the beginning of the surgical procedure, the mouth of the patient is scrubbed with either Betadine or, in case of intolerance, octenidin. Saliva surrounding the surgical site was continuously removed using an aspirator. In order to obtain a non-superficial specimen of the necrotic bone, the superficial layer of necrotic bone was removed using an ultrasonic bone cutting system. Then, a sample of necrotic bone was harvested, carefully avoiding contamination of the specimen by saliva, surrounding tissue, or contaminated medical instruments.

The bone specimens were subjected to microbiological examination using routine culture methods at the Institute for Medical Microbiology of the University Medical Center Eppendorf. In brief, tissue samples were homogenized and streaked onto Columbia blood agar, chocolate agar, and Schaedler agar (all Thermo Fisher, Bremen, Germany), and incubated at 37 °C in the presence of 5% CO_2_ and anaerobic, respectively. Plates were read every 48 h for a total of 14 days. Growing bacteria were differentiated to the species level using whole-cell mass spectrometry (Biotyper, Bruker, Bremen, Germany). Species known to potentially carry relevant acquired resistance markers (i.e., *Enterococcus* spp. [vanillate demethylase complex (*vanAB)*], *Staphylococcus aureus* [*mecA-gene*], Enterobacteriaceae [e.g., extended spectrum ß-lactamase (ESBL), carbapemases], *Pseudomonas aeruginosa*) were subjected to susceptibility testing using a Vitek II system (Biomerieux, Marcy L´Étoile, France). For species belonging to the resident flora of the oral cavity, susceptibilities were deduced from species identification.

### Statistical analysis

Data collection and analysis illustrations were done using Microsoft^®^ Excel^®^ and PowerPoint^®^ (Office 365^®^) and GraphPad Prism Version 4.03.

## Results

### Bacterial infection of bone is diverse, and simultaneous presence of different bacteria is common

**Fig. 1:**
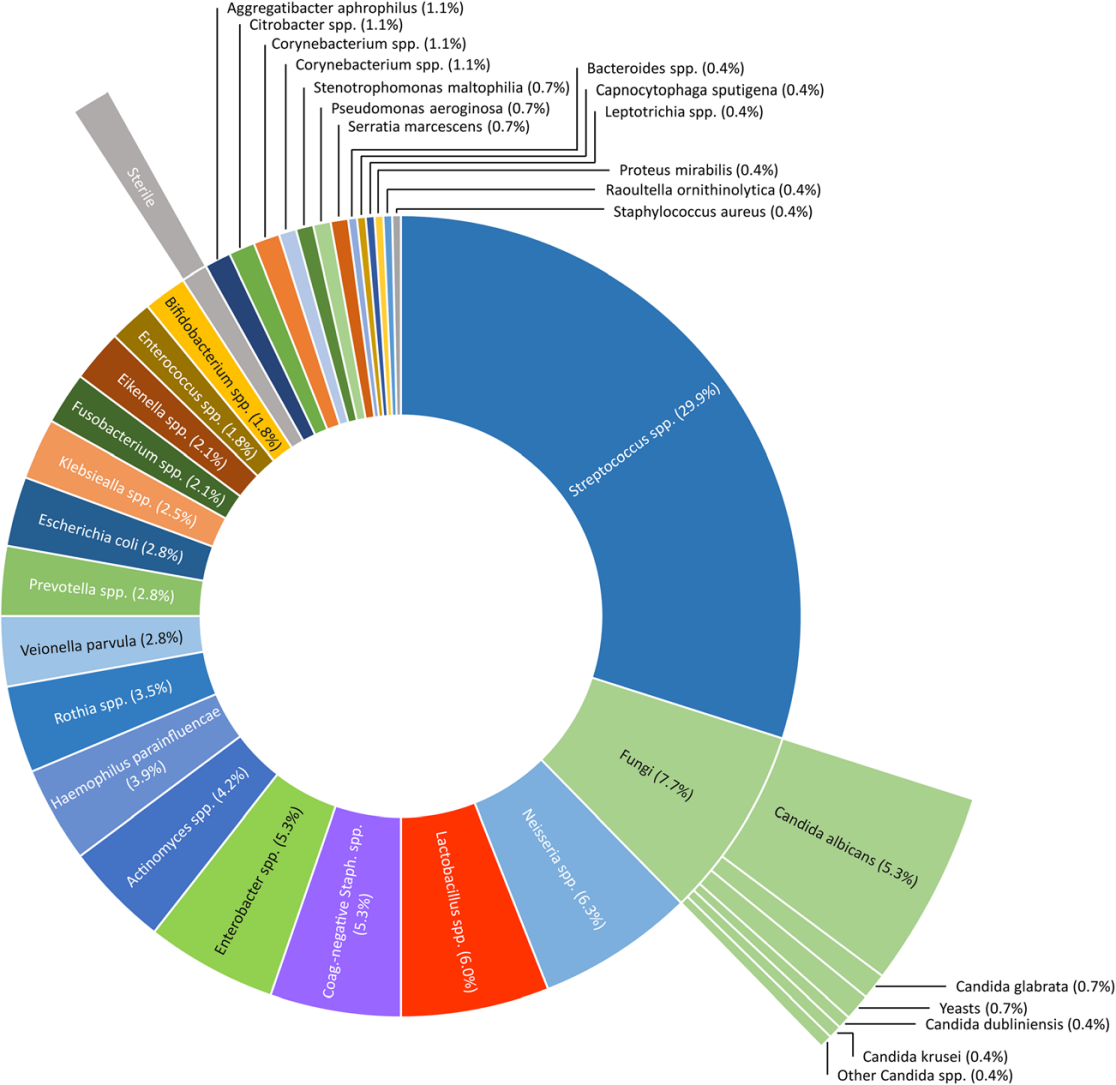
Overview over bacterial species and yeasts found in necrotic bone specimen. Pie chart of all bacterial genera and yeasts detected in the 116 specimens of necrotic bone. In three cases, no microbial colonization could be detected (indicated as sterile)

In the 116 specimens of necrotic bone, the presence of 43 different bacterial genera (a total of 259 isolates were found and, of these, 199 were identified to species level) and 6 different fungal species were detected using bacterial culture and whole-cell mass spectrometry (Fig. 1).

**Fig. 2:**
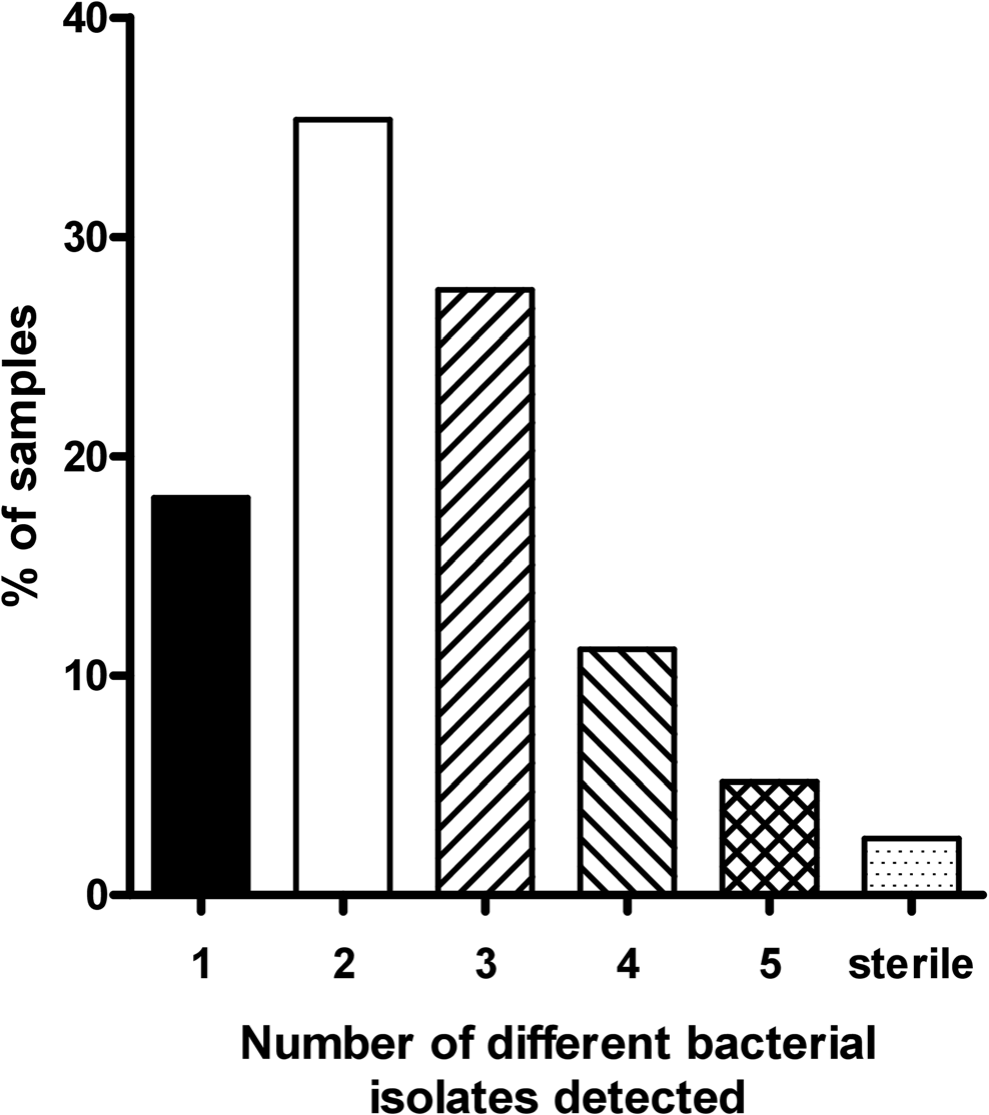
Number of different bacterial species or yeasts detected in necrotic bone specimen. In 18% of the 116 cases, the presence of one bacterial of fungal species was found, whereas in 79% of cases, two or more species were detected (i.e., 2 different species were detected in 35%, 3 in 28%, 4 in 11%, and 5 in 5% of cases, respectively). In three cases, no microbial colonization could be detected (indicated as sterile)

Cultivation revealed no bacterial growth in three cases. In 18% of cases, the presence of one bacterial of fungal species was found, whereas in 79% of cases, two or more species were detected (i.e., 2 different species were detected in 35%, 3 in 28%, 4 in 11%, and 5 in 5% of cases, respectively) (Fig. 2).

**Fig. 3:**
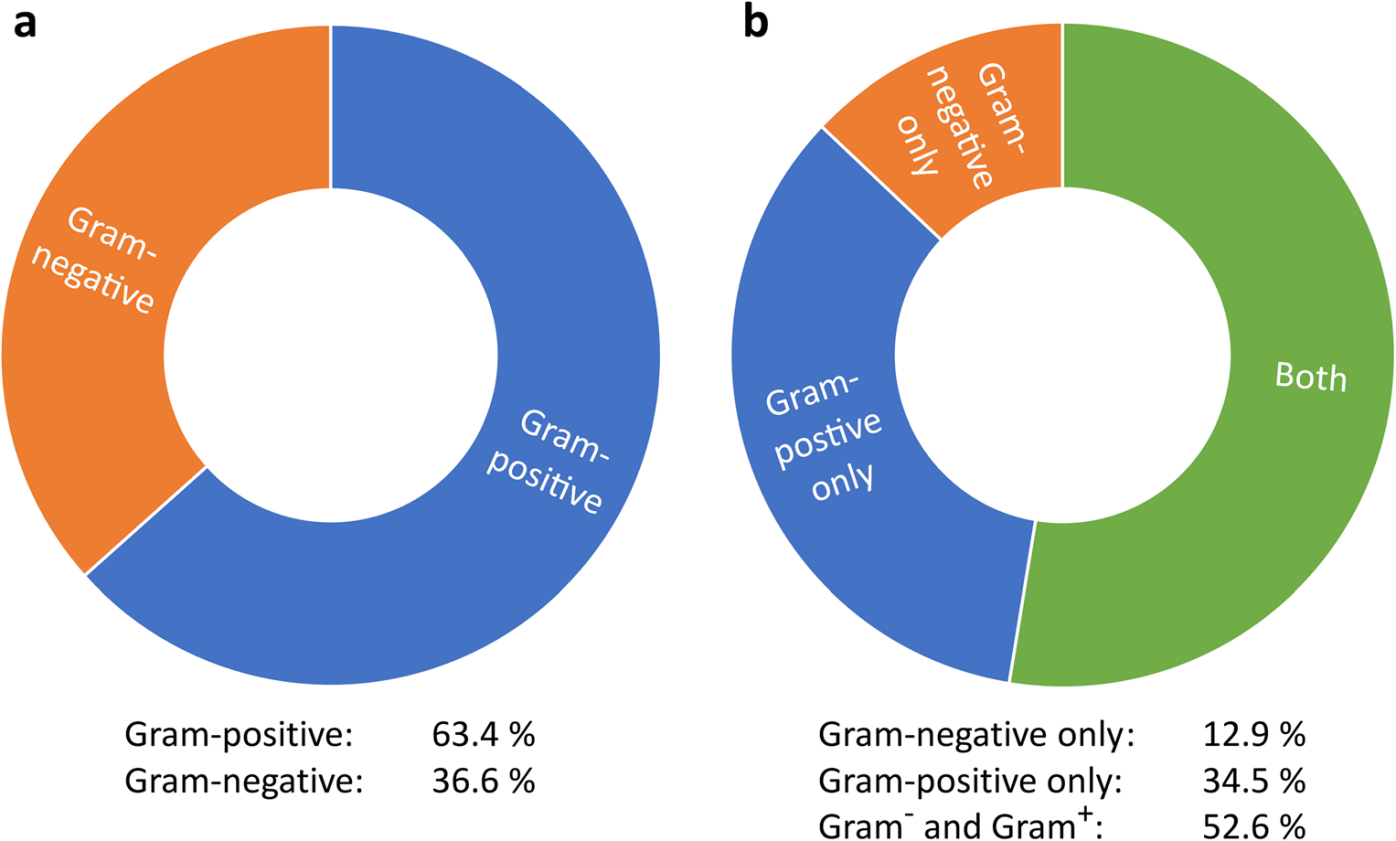
Distribution of gram-negative and gram-positives isolates. 63% of all isolates were gram-positive (**a**). However, at least one gram-negative isolate was detected in 65% of all samples analyzed (**b**)

Most frequently, *Streptococcus* spp., *Neisseria* spp., *Lactobacillus* spp., and coagulase-negative *Staphylococcus* genera were detected, accounting for about 60% of all isolates (Table 2). In total, 63% of all bacterial species detected were gram-positive (Fig. 3a). However, in 65% of all bone samples, at least one gram-negative isolate was identified, i.e., in 52% both gram-positive and gram-negative bacteria were present, and in 13%, gram-negative isolates only (Fig. 3b). The presence of yeast was detected in 25 of the 116 cases (21.5%).

**Table 2:** Overview over bacterial and fungal isolates found in necrotic bone specimen

Species	*n*	%
*Actinomyces* spp.	12	4.2
*Aggregatibacter aphrophilus*	3	1.1
*Bacteroides* spp.	1	0.4
*Bifidobacterium* spp.	5	1.8
*Capnocytophaga sputigena*	1	0.4
*Citrobacter* spp.	3	1.1
*Corynebacterium* spp.	3	1.1
*Eikenella* spp.	6	2.1
*Enterobacter* spp.	15	5.3
*Enterococcus* spp.	5	1.8
*Escherichia coli*	8	2.8
*Fusobacterium* spp.	6	2.1
*Haemophilus parainfluencae*	11	3.9
*Klebsiealla* spp.	7	2.5
*Lactobacillus* spp.	17	6.0
*Leptotrichia* spp.	1	0.4
*Neisseria* spp.	18	6.3
*Prevotella* spp.	8	2.8
*Proteus mirabilis*	1	0.4
*Pseudomonas aeroginosa*	2	0.7
*Oral flora, not specified*	2	0.7
*Raoultella ornithinolytica*	1	0.4
*Rothia* spp.	10	3.5
*Serratia marcescens*	2	0.7
*Staphylococcus aureus*	1	0.4
*Staphylococcus* spp*. coagulase-negative*	15	5.3
*Stenotrophomonas maltophilia*	2	0.7
*Streptococcus* spp.	85	29.9
*Veionella parvula*	8	2.8
*Yeasts*		
*Candida albicans*	15	
*Candida* spp.	1	
*Candida glabrata*	2	
*Candida dubliniensis*	1	
*Candida krusei*	1	
Other yeasts	2	
No bacterial colonization detected (“sterile”)	3

### Microbial flora changes over time in most patients

Fifteen patients underwent surgery for MRONJ twice in the same location, and 2 patients three times, with a mean interval between two surgeries of 103 days. The number of bacterial genera found in the respective bone specimen is presented in Table S2. In 40% of these cases, at least one bacterial genus was found in both consecutive bone samples. In the remaining cases, a different microbial flora, not present in the sample before, was observed.

### A high rate of β-lactamase inhibitor resistance can be observed

**Fig. 4:**
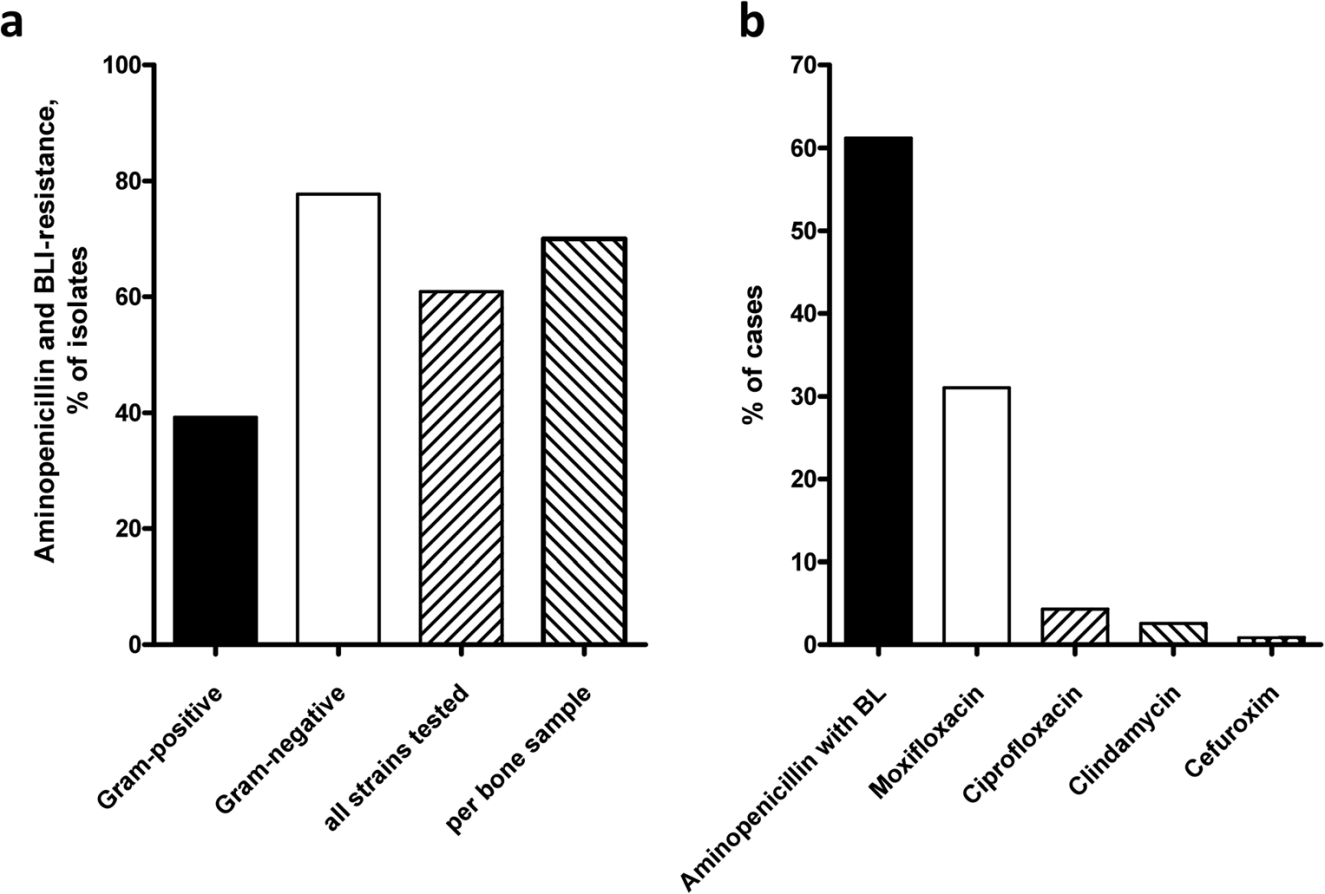
High rate of penicillin resistance among gram-negative isolates. A Penicillin resistance was observed in 39% of gram-positive and 78% of gram-negative genera, respectively. Of all isolates tested, 61% were found to be resistant against penicillin, and in 70% of all samples, at least one resistant species was detected. b Overview over initial antibiotic regimen in MRONJ patients; amoxicillin and clavulanic acid were administered twice daily; oral moxifloxacin was used in a once-daily regimen

For 67 bacterial isolates cultivated from necrotic bone, the corresponding susceptibility testing was available. Of the gram-positive bacteria, only about one-fourth was found to be resistant against penicillin, whereas almost 80% of gram-negative bacteria showed no susceptibility against the combination of ampicillin and a β-lactamase inhibitor (BLI) (Fig. 4a). Considering that in most cases more than one bacterial species was present in the necrotic bone, at least one penicillin-resistant species was observed in 70% of cases. Resistance against antibiotics of the fluoroquinolone family was only detected in 2 out of 38 isolates (5%).

### Effect of susceptibility testing on antibiotic treatment

In our study, amoxicillin and clavulanic acid were administered orally twice daily as the initial antibiotic regimen in about two-thirds of all patients (61%) (Fig. 4b). In about one-third of patients (31%), oral moxifloxacin was used in a once-daily regimen.

**Fig. 5:**
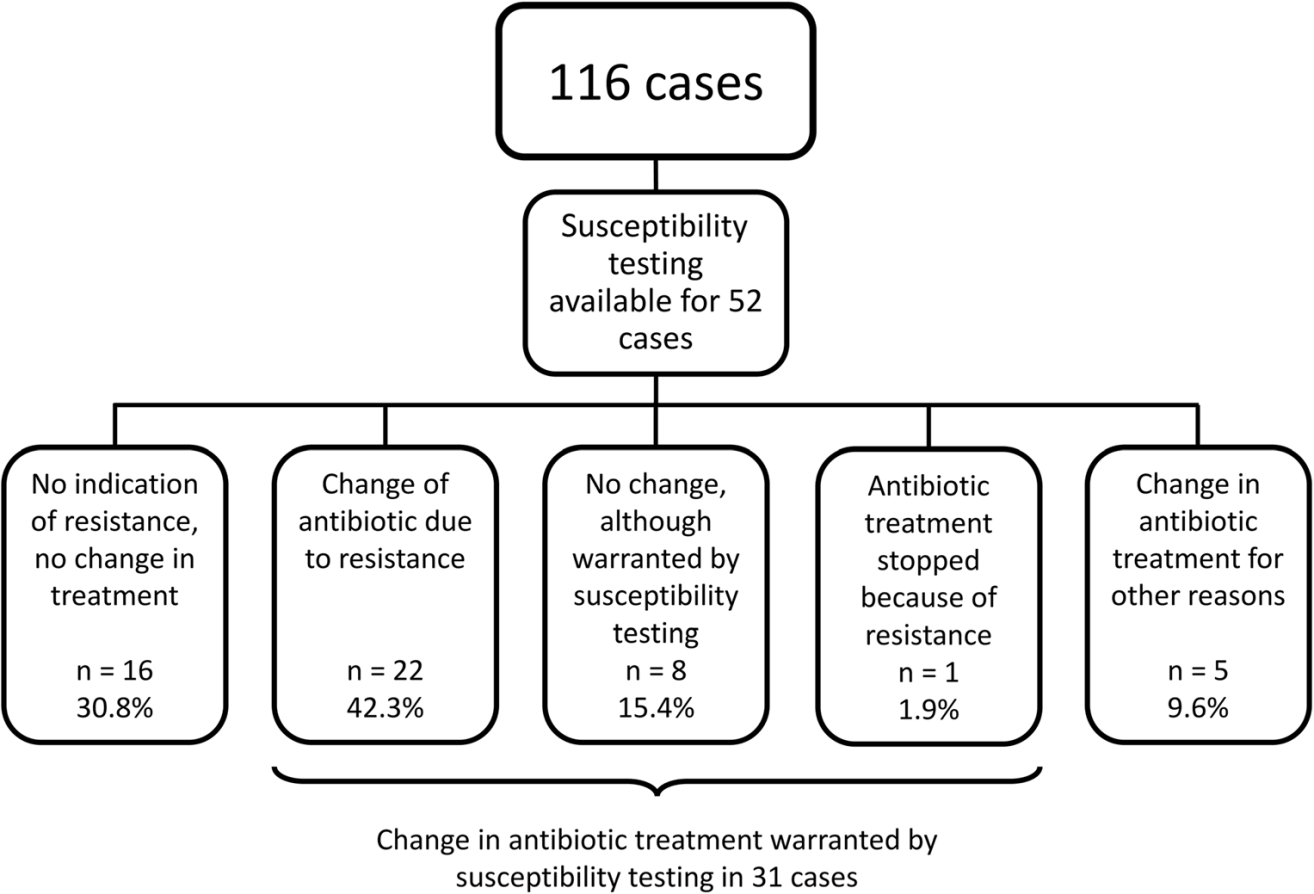
Influence of susceptibility testing on antibiotic treatment in MRONJ patients. Results from susceptibility testing were available for 52 of 116 cases. This led to changes in the antibiotic treatment in 22 of the 52 patients, whereas the treatment was continued in 16 patients. In 8 patients, although the presence of resistant bacteria was detected, no changes to the antibiotic treatment were made (e.g., because results from the susceptibility testing were overlooked). In one patient treated with moxifloxacin and the presence of resistance, the treatment was discontinued. In 5 patients, the antibiotic regimen was changed for other reasons, e.g., intolerance

Antibiotic treatment was continued until suture removal after 14 days. Therefore, when susceptibility testing was available for 52 patients, a change of the antibiotic regiment was warranted in 31 patients. Most of these patients (*n* = 27) were treated with amoxicillin and clavulanic acid, 2 were treated with moxifloxacin, and 1 patient was treated with oral clindamycin or cefuroxime, respectively. In most cases of penicillin resistance, moxifloxacin was used as second-line therapy. As shown in Fig. 5, results from susceptibility testing influenced the antibiotic treatment of 22 patients. However, in 8 patients, no changes to the antibiotic treatment were made, either because the patient did not appear for follow-up examinations, or results from the susceptibility testing were missed. In one patient treated with moxifloxacin and the presence of resistance, the treatment was stopped. In 5 patients, the antibiotic regimen was changes for other reasons, e.g., intolerance.

### Changes of the microbial composition over time in MRONJ patients treated for recurrent disease

Seventeen patients had recurrence of MRONJ, requiring surgical intervention; three patients required surgical treatment for a third time. Therefore, 37 bone specimens were available for microbiological examination. The mean interval between surgery was 103 days, and in 15 of the 17 patients, recurrent disease involved the same location. In 9 of the 17 cases, the microbial composition found in the necrotic bone specimens differed. In 8 cases, one or two bacterial or fungal genera were present in both bone specimen. In total, of the 75 isolates found, only 9 were present in all sequential bone samples of the respective patient (Figure S1A and S1B).

## Discussion

In recent treatment guidelines, there is a consensus that prolonged antibiotic treatment is indicated in MRONJ patients with signs of infection, i.e., in all patients with MRONJ stage 2 or 3 [11, 18, 20].

However, there is little guidance regarding the choice of antibiotics for initial empiric therapy in these patients. To our knowledge, a comprehensive analysis of the microbiome in necrotic bone considering susceptibility testing and antibiotic resistance has not been done yet.

To determine the spectrum of bacteria found in necrotic bone, a deep biopsy of affected bone was obtained, and routine culture techniques were applied. Other approaches, e.g., the detection of ribosomal RNA, may offer a more comprehensive coverage of bacteria present in tissues from polymicrobial infections. However, the culture method was chosen to determine the viable flora in the necrotic bone, and to allow for susceptibility testing.

During the process of harvesting the bone samples, considerable effort was done to avoid contamination. However, we cannot rule out that minimal contamination for example by aerosol formation or medical instruments contaminated by saliva from the adjacent oral cavity may have occurred. Furthermore, handling of the bone biopsy may have affected the detection of certain bacterial species, e.g., anaerobic bacteria, and the culture methods applied may be unsuited for the detection of some species.

Nevertheless, when comparing the microbial flora in biofilms from affected bone in MRONJ patients described by de Bruyn et al. using rRNA profiling with our results, there is a considerable similarity to the microbial flora found in our study. This includes the detection of bacterial genus associated with MRONJ development like *Aggregatibacter actinomycetemcomitans*, *Prevotella* spp., Fusobacterium or *Capnocytophaga* species, *Streptococcus mitis*, *Streptococcus gordonii*, *Actinomyces odontolyticus*, and *Veillonella* species [15, 16, 22].

In previous studies on the microbiome in MRONJ patients, an important role of *Actinomyces* species in the establishment of biofilms has been proposed. *Actinomyces* species could be detected in about 70% of samples from necrotic bone using histological techniques [15, 23–25]. In this study, we have detected *Actinomyces* species only in 12 of the 116 samples (10.3%). This disparity might be due to the technical challenges in the cultivation of *Actinomyces* species [26], consistent with results from Panya et al., showing a higher sensitivity for PCR in detecting *Actinomyces* species [23].

Our data highlight that colonization of necrotic bone by gram-negative bacteria is frequent. Gram-negative bacteria are known to have a high probability of intrinsic or acquired resistance toward penicillin [27]. We were able to identify bacterial isolates harboring aminopenicillin and BLI resistance in 70% of all patients. However, this result may have been biased by the fact that all patients received at least 1 week of antibiotic treatment before surgery, possibly causing an imbalance in the oral flora and providing gram-negative bacteria with a selective advantage. As antibiotic treatment is warranted to be started before invasive procedures take place, we were unable to include an antibiotic-naïve control group in our study.

Our study highlights the importance of susceptibility testing, as recommended by the American Association of Oral and Maxillofacial Surgeons [18]. Furthermore, our results give important advice considering the choice of antibiotics for initial treatment of MRONJ patients. Since most patients are treated in the outpatient setting, antibiotic treatment should not only be effective against gram-negative bacteria, but also provide a good oral bioavailability. Therefore, we now routinely use fluoroquinolones (i.e., moxifloxacin or ciprofloxacin) instead of penicillin antibiotics in MRONJ patients with stage 2 or 3 disease. However, the use of fluoroquinolones can have severe side effects, especially in older patients and those with multiple morbidities. Using routine susceptibility testing may help to avoid using fluoroquinolones in cases where it may not be necessary.

## Conclusion

If antibiotic treatment is warranted in MRONJ patients, the empiric choice of antibiotics should consider the high rate of gram-negative bacteria, or cultivation methods should be used to help guide the antibiotic treatment. The common application of antibiotics, especially clindamycin or amoxicillin in dental or oral surgical procedures, may lead to an increasing frequency of bacterial resistance. This can become a serious problem, especially in patients with MRONJ, osteoradionecrosis, or other infectious diseases of the bone. Therefore, antibiotic treatment should be reconsidered in each case and each patient. Especially in MRONJ patients, an effective antibiotic therapy might reduce the risk for wound healing disorders resulting in recurrence of necrotic bone areas. Therefore, further research is warranted for the evaluation and development of potentially more rational antibiotic therapies, with a special emphasis on the efficient delivery of antibiotics to the hypovascular bone matrix.

## Electronic supplementary material

Figure S1 Changes of the microbial composition over time in patients with recurrent disease. (A) Overview over the number of different bacterial or fungal isolates observed in necrotic bone samples obtained during the first and second surgical treatment. (B) Comparing the microbial composition of bone samples from patients with recurrent disease reveals that only few bacterial isolates were present at both time points. (PNG 167 kb)

High Resolution Image (TIFF 101928 kb)
