# Supplementary figures and images for: Retrospective analysis of bacterial colonization of necrotic bone and antibiotic resistance in 98 patients with medication-related osteonecrosis of the jaw (MRONJ)
Source: Clin Oral Investig. 2020 Oct 2;25(5):2801–9. doi: 10.1007/s00784-020-03595-9 (PMC8060223; doi:10.1007/s00784-020-03595-9)

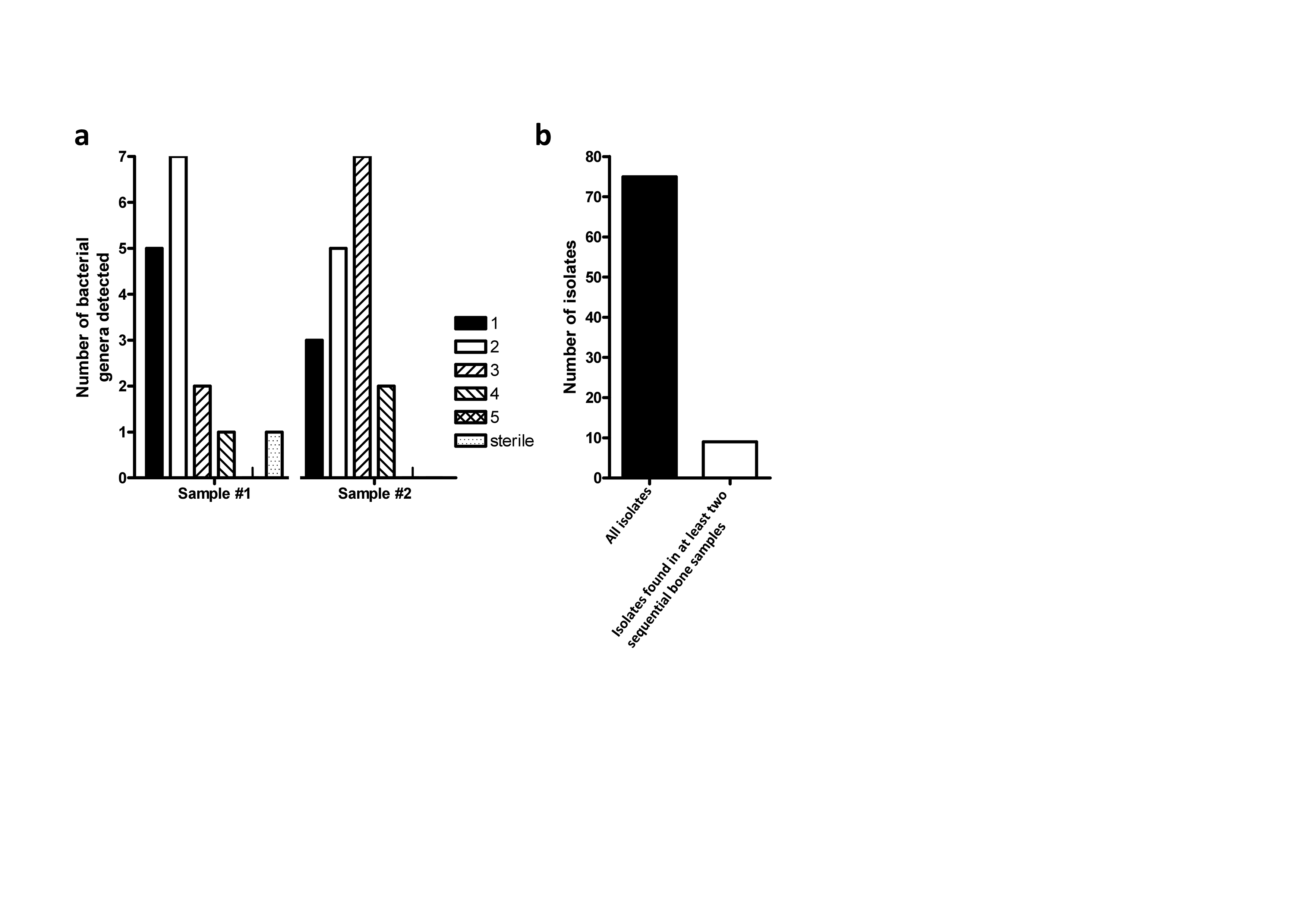

Supplement: Supplementary file 1 — Changes of the microbial composition over time in patients with recurrent disease. (A) Overview over the number of different bacterial or fungal isolates observed in necrotic bone samples obtained during the first and second surgical treatment. (B) Comparing the microbial composition of bone samples from patients with recurrent disease reveals that only few bacterial isolates were present at both time points. (PNG 167 kb) [file 784_2020_3595_Fig6_ESM.png]
